# Effect of Physical Training on Body Composition in Brazilian Military

**DOI:** 10.3390/ijerph19031732

**Published:** 2022-02-02

**Authors:** Luis Alberto Gobbo, Raquel David Langer, Elisabetta Marini, Roberto Buffa, Juliano Henrique Borges, Mauro A. Pascoa, Vagner X. Cirolini, Gil Guerra-Júnior, Ezequiel Moreira Gonçalves

**Affiliations:** 1 Skeletal Muscle Assessment Laboratory (LABSIM), School of Technology and Science, São Paulo State University (UNESP), Presidente Prudente 19060-900, SP, Brazil; luis.gobbo@unesp.br; 2 Growth and Development Laboratory, Center for Investigation in Pediatrics (CIPED), School of Medical Sciences, University of Campinas (UNICAMP), Campinas 13083-887, SP, Brazil; raqueldlanger@gmail.com (R.D.L.); borgesedfisica@gmail.com (J.H.B.); pascoawaf@bol.com.br (M.A.P.); vxcirolini@hotmail.com (V.X.C.); gilguer@fcm.unicamp.br (G.G.-J.); emaildozeique@gmail.com (E.M.G.); 3 Department of Life and Environmental Sciences, University of Cagliari, 09042 Monserrato, Italy; rbuffa@unica.it

**Keywords:** bioelectrical impedance, vector analysis, lean soft tissue, fat mass, muscle mass, phase angle

## Abstract

The military are selected on the basis of physical standards and are regularly involved in strong physical activities, also related to particular sports training. The aims of the study were to analyze the effect of a 7-month military training program on body composition variables and the suitability of specific ‘bioelectrical impedance vector analysis’ (spBIVA), compared to DXA, to detect the changes in body composition. A sample of 270 male Brazilian cadets (19.1 ± 1.1 years), composed of a group practicing military physical training routine only (MT = 155) and a group involved in a specific sport training (SMT = 115), were measured by body composition assessments (evaluated by means of DXA and spBIVA) at the beginning and the end of the military routine year. The effect of training on body composition was similar in SMT and MT groups, with an increase in LST. DXA and spBIVA were correlated, with specific resistance (Rsp) and reactance (Xcsp) positively related to fat mass (FM), FM%, LST, and lean soft tissue index (LSTI), and phase angle positively related to LST and LSTI. Body composition variations due to physical training were recognized by spBIVA: the increase in muscle mass was indicated by the phase angle and Xcsp increase, and the stability of FM% was consistent with the unchanged values of Rsp. Military training produced an increase in muscle mass, but no change in FM%, independently of the sample characteristics at baseline and the practice of additional sports. SpBIVA is a suitable technique for the assessment of body composition in military people.

## 1. Introduction

The military paradigm is associated with healthy appearance, athletic bearing, and high-level physical performance. Indeed, the military are selected based on physical standards and are regularly involved in strong physical activities, also related to sports training, which requires monitoring for variations in body composition variants [1].

There are various methods usable to evaluate body composition, including anthropometry; bioimpedance; and more accurate techniques, such as potassium 40 counting, water isotope dilution, underwater weighing, imaging techniques, and dual energy X-ray absorptiometry (DXA) [2].

Due to the high suitability and low cost, the anthropometric techniques are the most used in many fields of application, including the routine military practice [3,4,5]. These methods, however, are not very accurate in detecting the main body compartments. For example, body mass index does not distinguish lean mass from fat mass [6,7,8] and so is incapable of evaluating muscle mass gain concomitant to fat weight loss (as generally occurs with intense military training) [9]. Accordingly, Pierce et al. [10] have recently demonstrated that BMI is not associated with performance on military relevant tasks in U.S. Army soldiers. Further, waist circumferences, largely used among the military [3,4,5] due to the associations with intra-abdominal fat and the related morbidity outcomes [11], are subject to intra- and inter-observer errors of measurement [12] and the need for a strong standardization because of the different possible measurement sites. Research results in military members are discordant, showing both a good and a poor agreement between the circumference measurement body composition method and dual-energy X-ray absorptiometry (DXA) [5,13].

Bioelectrical impedance analysis (BIA) is a non-invasive, low cost, and easy to operate technique, which needs a very short time compared to the more sophisticated body composition methods [14]. BIA has been rarely applied to research in the military showing a good agreement with DXA results [13,15,16]. The traditional two-component approach of BIA uses predictive equations, including bioelectrical values (generally resistance), and considers other variables (age, sex, and height) for the evaluation of fat mass and fat-free mass [17]. However, the application of predictive equations in samples differing from those where they have been calibrated can introduce a source of error. Otherwise, the use of population/group-specific equations reduces the comparability of results.

Alternative approaches, that have been proposed to avoid the use of equations and possible related errors, are based on the analysis of raw bioelectrical data of resistance (R, ohm) and reactance (Xc, ohm). The phase angle (arctan Xc/R 180/π, degrees) is an indicator of nutritional status related to body cell mass and cell membrane integrity, that is largely used in clinical practice [18,19]. Phase angle has also been analyzed in relation to resistance training, and an increasing trend of its values has been registered [20]. However, as shown by Mereu et al. [21], the analysis of body composition based on the phase angle only can be inaccurate and is significantly improved if the information given by the vector length (R^2^ + Xc^2^)^0.5^ is also considered.

Such a vectorial approach has been proposed by Piccoli et al. [22], who conceived the bioelectrical impedance vector analysis (BIVA). The classic BIVA procedure analyzes the bioelectrical values of resistance and reactance, standardized for body height (a proxy of conductor length). A BIVA variant defined as ‘specific bioelectrical impedance vector analysis’ (spBIVA) implies the standardization of resistance and reactance by length and by cross-sections of the body as well [22,23,24]. SpBIVA has been shown to be effective in the evaluation of fat mass percentage [23,24,25] and skeletal muscle mass [9,23,26,27]. Specific reference values have been proposed for 50 different populations, such as Italian-Spanish, U.S. young adults, and Italian healthy elderly [22,27,28]. The classic BIVA approach has been sporadically used in relation to sport and exercise [29], and specific BIVA even less [29,30,31,32]. Neither classic nor specific BIVA has been applied to the military samples.

The aims of the present study were two-fold: (1) to analyze the effect of a 7-month military training program on body composition variables, and (2) to analyze the correlation between the changes in body composition measured by spBIVA and DXA in a Brazilian military sample.

## 2. Materials and Methods

In accordance with the Helsinki Declaration, a written informed consent was obtained from all participants. The research was approved by the Ethics Committee of the School of Medical Sciences, University of Campinas. All procedures followed Resolution No. 466 of 2012 of the National Health Council of the Ministry of Health of Brazil.

### 2.1. The Sample

A sample of 270 young men (19.1 ± 1.1 years) from all the regions of Brazil (South, Southeast, Midwest, North, and Northeast) enrolled in the Preparatory School of Army Cadets (EsPCEx) of the city of Campinas, SP, Brazil, was selected. Data were collected over two years (2013 and 2014), in two periods: at the beginning (March/April) and the end (October/November) of the military routine year.

The sample was divided into two groups: (1) the cadets who were involved in the military physical training routine only (MT, n = 155); (2) the cadets who were involved, by their own choice, in the military physical training routine plus a specific sport training for military competition (SMT, n = 115): track and field (n = 25), basketball (n = 16), fencing (n = 3), soccer (n = 18), judo (n = 3), swimming (n = 13), trekking (n = 4), shooting (n = 2), triathlon (n = 11), volleyball (n = 15), or chess (n = 5).

All cadets were included in the sample, except those who did not sign the consent form, who did not attend the day of the evaluations (even if only the second ones), who had a history of musculoskeletal injury at the time of the assessments, or were disconnected from the school.

### 2.2. Military Physical Training

Military physical training was performed 5 days/week during 90 min/day for 34 weeks, according to the academy military physical training manual, where the cadets were supposed to undergo a physical training that consisted of: (a) 2–3 sessions/week of continuous or interval running, with a weekly increased load; (b) 1 session/week of calisthenics exercises (7–15 repetitions of push-up, push-up/stand-up, sit-up, squat with hands on hip, lunge with hands on hip, and jumping jacks); (c) 1–2 sessions/week of circuit resistance training (2 sets of bench press, sit-up and its variations, half squat, barbell curl, pull-up, stair jumps, jump rope, and wrist roller, with 30 s of each exercise and 30 s of rest interval); (d) 1 session/week of swimming; and (e) 2 sessions/week of sports training. Before each session, all participants went through ~8 stretching exercises, ~7 neuromuscular warm-up, and ~7 general warm-up exercises. For sports training, each participant performed specific training for each modality [32,33].

### 2.3. Measurements

All subjects underwent anthropometric, BIA and DXA assessments, in the same sequence, in the morning.

Anthropometric measurements were performed following standard procedures [34], by an accredited International Society for the Advancement of Kinathropometry (ISAK) technician. Body weight (kg) and height (cm) were measured using a digital scale with precision of 0.1 kg (Filizola, São Paulo, Brazil) and a Harpenden stadiometer with precision of 1 mm (Holtain Limited, Crosswell, UK), respectively. Relaxed upper arm, waist and calf girths were measured using an anthropometric tape (precision of 1 mm). Body mass index in kg·m^−2^ was calculated by the ratio between body weight, in kilograms, and height squared, in meters (BMI).

A fan beam equipment model iDXA (GE Healthcare Lunar, Madison, WI, USA), enCore^tm^ 2011 software (version 13.6), was used to determine body composition. Total body composition was measured with the subject lying in the supine position, with the scanning time for the full length of approximately seven minutes. Total fat mass (FM, kg), fat percent (%FAT), lean soft tissue (LST, kg), and bone mineral content (BMC, kg) were measured. LSTI (kg·m^−2^) was calculated as LST/height squared in meters. To determine the reproducibility of the variables estimated by the equipment, coefficient of variation (CV%) and the technical error of measurement (TEM) were determined, based on the testing and retesting conducted with 23 subjects, and retested within 24 h. The values of CV% were 0.74%, 0.28%, and 0.26% for FM, BMC, and LST, respectively, and TEM were 0.25 kg (FM), 0.02 kg (BMC), and 0.25 kg (LST).

Bioelectrical measurements (resistance (R), ohm; reactance (Xc) ohm; at 50 kHz and 425 μA) were taken following the standard procedure [14]. With a Bioelectrical Body Composition Analyzer, tetrapolar device, single frequency (50 kHz), and model Quantum II (RJL Systems, Detroit, MI, USA). Specific bioelectrical impedance vector analysis was applied [24]. Specific bioelectrical values (resistivity (Rsp) ohm cm; reactivity (Xcsp) ohm cm) were obtained by multiplying resistance and reactance by a correction factor (A/L), where area (A, cm^2^) and length (L, cm) were estimated as follows: A = (0.45 upper arm area + 0.10 waist area + 0.45 calf area) and L = 1.1 stature (in cm). The segment areas were calculated as C2/4π, where C (cm) is the girth of the upper arm, waist, or calf. The phase angle (degrees) was calculated as arctan (Xc/R 180/π) and the impedivity vector (Zsp, ohm cm) as (Rsp^2^ + Xcsp^2^)^0.5^.

All participants should have fasted for at least 4 h, not ingest caffeinated foods or alcoholic beverages 24 h prior to the test, not perform strenuous physical activity less than 12 h before the test, not use any diuretics for at least 7 days before the test, urinate about 30 min before the test, and remove all metals (bracelets, watch, chains, etc.). During the assessment, the volunteers remained in the supine position, on a stretcher isolated from electrical conductors, in the supine position, with the legs abducted at an angle of approximately 45 degrees [35]. The values of CV% were 0.35% and 0.33% for R and Xc, respectively, and TEM were 3.54 Ω and 0.49 Ω, respectively, for R and Xc, for the same 23 subjects retested for DXA. Gonzalez et al. [36] validated the same equipment used in this study in a Brazilian sample, also using DXA as a reference method.

The within-sample variability was investigated by considering the distribution of bioelectrical values in the tolerance ellipses, representing the bivariate percentiles of the reference population. At this purpose, the tolerance ellipses for the Italo-Spanish adults (18–30 years) [28] have been used. The major axis of the ellipses refers to variations of FM% (higher values towards the upper pole) and the minor axis to variations of skeletal muscle mass and ECW/ICW (lower values on the left side).

### 2.4. Statistical Analyses

Bioelectrical values of MT and SMT groups were compared by mean of tolerance and confidence ellipses, using a two-sample Hotelling’s T^2^ test.

The consistency of the results obtained with specific BIVA and DXA was evaluated by means of Pearson’s correlation between bioelectrical and DXA variables at baseline and comparing the trend of longitudinal body composition variations described by the two techniques.

The effect of training (pre- vs. post-training) in the two sub-samples of SMT and MT was analyzed using two-way ANOVA (anthropometric, DXA output and bioelectrical values) and paired one-sample Hotelling’s T^2^ (confidence ellipses).

Statistical analyses were performed using IBM SPSS Statistics 19 (IBM SPSS Statistics for Windows, Version 19.0. Armonk, NY: IBM Corp) and the specific BIVA software (freely available at the website: http://specificbiva.unica.it/ (accessed on 17 August 2018).

## 3. Results

On average, at baseline, the sample of military people was in the normal weight BMI category and had a percent fat mass within the normal range for men (Table 1).

**Figure 1:**
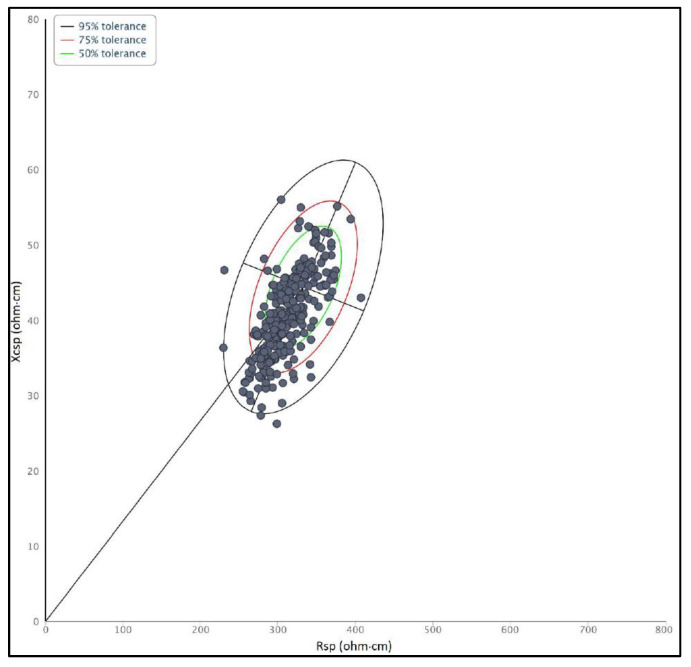
Distribution of bioelectrical values of Brazilian Military onto tolerance ellipses representing Italian-Spanish young adults, at the beginning of the routine year.

Bioelectrical values were quite totally within the reference tolerance ellipses, but slightly shifted toward the lower pole, indicative of low FM% (Figure 1).

DXA and specific BIVA were correlated (Table 2). In fact, Rsp and Xcsp were positively related to FM, FM%, LST, and LSTI, while phase angle was positively related to LST and LSTI.

**Figure 2:**
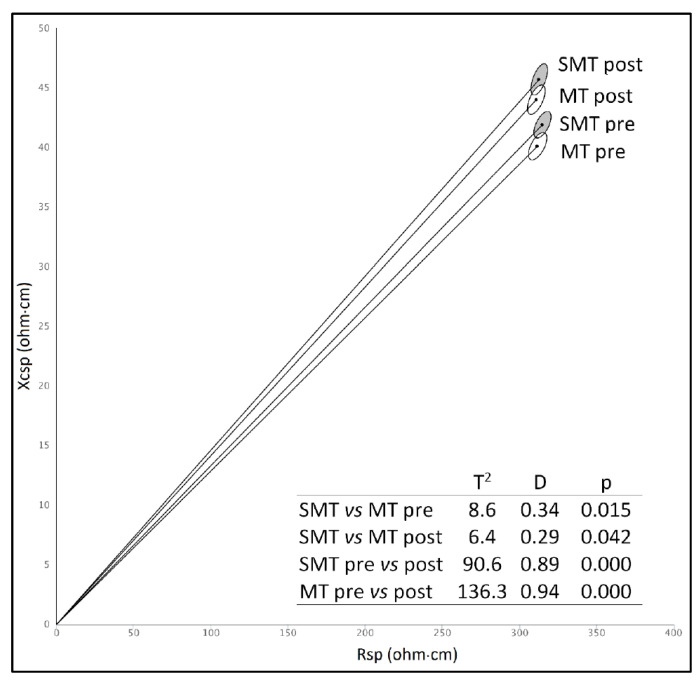
Confidence ellipses with T^2^ Hotelling’s test in the two groups before and after training. Legend: SMT: Sports and Military Training; MT: Military Training only. Comparisons between SMT and MT were performed using two-sample Hotelling’s T2 tests, while those between pre- and post- training groups were performed with paired one-sample Hotelling’s T^2^ tests.

Military people practicing sport activities (SMT) showed body composition differences with respect to those practicing military training only (MT). In fact, SMT group had higher values of LST and BMC, and lower values of FM and FM% (Table 3). The bioelectrical values of specific reactance and phase angle were significantly higher in SMT than in MT, indicating higher muscle mass (Table 3, Figure 2).

## 4. Discussion

In this sample of military personnel, DXA and specific BIVA showed a consistent scenario of body composition variations related to physical training. In fact, specific bioelectrical variables were correlated with DXA (FM, FM% and LST, and LSTI), and both the techniques showed: (a) different body composition in the military practicing physical training routine only (MT) or a specific sport training as well (SMT); (b) an increase in fat-free mass and a steady percentage of fat mass in relation to training, in both SMT and MT groups.

The sample, especially the SMT group, showed body composition characteristics adequate to the military standard, as suggested by the BMI indicative of normal weight [37], and the percentage of fat mass, which was lower than the body fat limits of approximately 20%, desirable for the U.S. army men [4]. The values of fat-free mass were higher in SMT than MT. The period of over ~7 months of military training induced, in both MT and SMT groups, a gain of lean soft tissue that contributed to the higher value of weight and BMI. However, the absolute and relative quantity of body fat did not change.

The observed differences of body composition are consistent with the effects of physical training described in the general population, and in the military [38,39]. Aerobic, stretching and resistance training are among the main interventions that can affect fat mass, fat free mass, and skeletal muscle mass, especially in young adults. These effects can be achieved in adults in a period of 3 to 12 months, depending on the characteristics of the sample and the volume of training, as well as other influent factors, such as daily habits and, particularly, alimentary style [40].

Research focused on military training has shown in general an increase in fat-free mass [15,16,34,41,42,43], but not in Margolis et al. [44], while the results on fat mass are less consistent among the studies. Mikkola et al. [16], in Finnish military performing regular, rather high-intensity, physical activity, over a period from 6 to 12 months, observed an increase in fat mass (in normal weight individuals), but a decrease in visceral fat. Indeed, intense physical activity promotes a greater reduction of visceral than subcutaneous adipose tissue, even if weight increases [45].

As previously presented, despite the inability of indicators such as BMI and waist circumference to validly identify lean and fat mass in physically active soldiers [6,7,8,9,10,11,12], there is still a gap in studies with DXA, for example, to test the agreement with the bioimpedance technique [5,13].

From a methodological point of view, similarly to the present research, previous studies realized in U.S. adults [9,23] and elderly Italians [24] detected a high correlation between DXA and specific BIVA variables. In particular, Rsp and Xcsp showed a positive correlation with FM% (especially Rsp; [9,24]) and with FFMI (especially Xcsp; [9]), while phase angle was positively related to FFMI only [9]. It is noteworthy that such convergent results have been obtained in samples characterized by different geographical provenience (Brazil, present study; US and Italy) [9,23,24], age class (Young adults, adults, and elders), and lifestyle (military, general population, and retirees). Indeed, the observed relationships are expected. In fact, resistance is negatively related to total body water and electrolytes, and hence, in normal-hydrated individuals, increases with the proportion of low conductive tissues, such as fat mass [9,24]. On the other side, the capacitive component (reactance) and phase angle are associated with the polarization produced by cell membranes and tissue interfaces and are positively related with body cell mass [22]. In this study, the correlation between DXA and spBIVA is shown by the trajectory of vector migration in relation to training, that, in both MT and SMT groups, is associated with increased values of reactance and phase angle (increasing muscle mass), but quite unchanged Rsp values (stable FM%). Such results can be comparable to those of Campa et al. [46], who analyzed three different sports modalities (volleyball, soccer, and rugby) and observed higher PhA values in athletes with a high mesomorphic component, which means, higher skeletal muscle component.

However, inconsistent results between DXA/spBIVA and waist circumference have been observed. In fact, SMT group showed higher values of waist circumferences, increasing in time, with respect to MT, but lower FM% levels, which remained stable after the military training. A similar disagreement between DXA and the circumference methods has already been described in the military [5]. In our research, we have also observed that spBIVA results, similarly to DXA, were not consistent with the pattern of waist circumference differences. However, compared to DXA, spBIVA did not recognize a lower percentage of fat mass in SMT with respect to MT. The observed gaps between abdominal circumference and DXA or spBIVA are noteworthy, considering the particular emphasis given to circumference measurement to calculate body fat percentage among the military [42]. The inconsistencies can be likely related to the different distribution of body components in the central and peripheral regions of the body and maybe to the greater effect of training on visceral than on subcutaneous fat, discussed above.

Despite the fact that the present study analyzed cadets who practiced 11 different sports, approximately 85% of the total SMT group practiced either teams’ sports or individual sports, such as cyclical sports (swimming, athletics, or triathlon), therefore, the physiological characteristics were not so different when comparing practitioners of sports modalities, such as basketball, with practitioners of judo or fencing. In this way, the variations in the subjects’ body composition, especially in the SMT group, are partly explained by training in some sports that total almost 90% of all the modalities practiced.

Considering, for example, the practice of team sports, such as soccer and volleyball, as was verified in different studies [47,48] that the longer the training time, the greater the phase angle and the lower the resistance values, indicating higher lean body mass values and, consequently, higher musculoskeletal mass. Micheli et al. [47] demonstrated that elite Italian professional football players (Series A and B) trained 9 weeks more throughout the year, with three more training sessions per week and one more game per week than amateur players, and consequently presented 7% higher and 8% lower phase angle and resistance values, respectively, indicating better body compositing status. In contrast, in our study, the differences between the phase angle (MT × SMT) were due to the greater values of reactance for those who practice sports activities. Such a situation can be explained by the amount of military training of both groups, which naturally provides good physical fitness, with lower resistance values. The SMT group, with specific sports training, presented higher values of phase angle, explained by higher reactance values, influenced mainly by greater amount of cell mass.

The main strengths of the present research are related to the application of a standardized protocol with cross-sectional and longitudinal measures, the use of reference techniques for body composition assessment in association with specific BIVA, and a well-controlled sample. However, some limitations are also present and are related to the poor representation of cases in the different disciplines, characterized by different training protocols, which made it impossible to recognize possible differences in body composition changes and in the underlying physiological mechanisms. Further, there was some disagreement between methods (anthropometry, DXA, and specific BIVA), likely related to regional differences of body components, which should be better analyzed by means of localized body composition analysis.

## 5. Conclusions

In conclusion, this research showed that spBIVA is a suitable technique for the assessment of body composition in the population studied. The effect of training on body composition was independent of sample characteristics or type of physical exercise: muscle mass increased, while the percentage of fat mass remained unchanged.

## Figures and Tables

**Table 1:** Descriptive statistics for body composition of Brazilian Military (N = 270) at the beginning of the routine year.

Variables	Mean	SD	95% IC (Lower–Upper Limits)
Weight, kg	69.9	8.9	68.9–71.0
Height, cm	175.7	6.4	174.9–176.4
BMI, kg·m^−2^	22.6	2.4	22.4–22.9
Waist crf, cm	76.2	4.8	75.7–76.8
LSTI, kg·m^−2^	17.9	1.6	17.7–18.0
FM, kg	12.2	3.7	11.7–12.6
LST, kg	55.2	6.4	54.4–55.9
BMC, kg	3.0	0.4	2.9–3.0
FM%	17.1	3.8	16.6–17.5
Rsp, ohm·cm	313.0	28.8	309.8–316.8
Xcsp, ohm·cm	40.9	5.7	40.2–41.6
Phase Angle, degrees	7.4	0.8	7.3–7.5

Legend: SD = standard deviation; BMI = body mass index; LSTI = lean soft tissue index; FM = fat mass; LST = lean soft tissue; BMC = bone mineral content; FM% = fat mass percent; Rsp = specific resistance; Xcsp = specific reactance.

**Table 2:** Matrix of correlation between bioelectric and DXA variables (N = 270) at baseline.

	Rsp		Xcsp		PA	
	r	*p*	r	*p*	r	*p*
FM, kg	0.582	0.000	0.406	0.000	0.030	0.627
FM%	0.556	0.000	0.326	0.000	−0.049	0.418
LST, kg	0.229	0.000	0.300	0.000	0.189	0.002
LSTI, kg·m^−2^	0.292	0.000	0.497	0.000	0.400	0.000

Legend: r = Pearson correlation coefficient; *p* = *p* value; FM = fat mass; FM% = fat mass percent; LST = lean soft tissue; LSTI = lean soft tissue index; Rsp = specific resistance; Xcsp = specific reactance; PA = phase angle.

**Table 3:** Descriptive and comparative statistics.

	SMT (N = 115)				MT (N = 155)						
	Pre		Post		Pre		Post				
	Mean	Sd	Mean	Sd	Mean	Sd	Mean	Sd	Fg	Ft	Fgxt
Weight, kg	71.0	8.7	73.0	8.7	69.1	9.0	70.8	8.2	0.006	0.018	0.826
Height, cm	176.4	6.3	176.7	6.2	175.1	6.5	175.3	6.5	0.023	0.521	0.979
BMI, kg·m^−2^	22.8	2.1	23.3	2.1	22.5	2.5	23.0	2.2	0.147	0.014	0.868
Waist crf, cm	77.2	4.9	78.5	5.1	75.5	4.6	77.2	4.3	0.000	0.000	0.581
FM, kg	11.5	3.2	12.2	3.1	12.6	4.0	12.7	3.3	0.011	0.253	0.305
LST, kg	56.9	6.6	58.1	6.6	53.9	5.9	55.5	5.8	0.000	0.009	0.671
LSTI, kg·m^−2^	18.3	1.6	18.6	1.5	17.6	1.5	18.0	1.4	0.000	0.004	0.541
BMC, kg	3.1	0.4	3.1	0.4	2.9	0.4	3.0	0.4	0.000	0.118	0.991
FM%, %	16.0	3.3	16.5	3.1	17.9	3.9	17.6	3.3	0.000	0.720	0.199
Rsp, ohm	314.8	27.6	312.7	26.5	311.7	29.6	310.8	28.3	0.316	0.544	0.816
Xcsp, ohm	41.9	5.5	45.7	6.5	40.1	5.7	44.0	6.1	0.001	0.000	0.911
PA, degree	7.6	0.8	8.3	0.9	7.3	0.7	8.1	0.8	0.001	0.000	0.966

Legend: SMT: Sports and Military Training; MT: Military Training only; F, F test of two-way ANOVA for group (Fg), training (Ft), and group-training interaction (Fgxt); BMI = body mass index; FM = fat mass; FM% = fat mass percent; LST = lean soft tissue; LSTI = lean soft tissue index; Rsp = specific resistance; Xcsp = specific reactance; PA = phase angle.
